# Willingness to Participate in Vaccine-Related Clinical Trials among Older Adults

**DOI:** 10.3390/ijerph15081743

**Published:** 2018-08-14

**Authors:** Divyanshu Raheja, Evelyn P. Davila, Eric T. Johnson, Rijalda Deović, Michele Paine, Nadine Rouphael

**Affiliations:** Hope Clinic of the Emory Vaccine Center, Division of Infectious Diseases, Department of Medicine, School of Medicine, Emory University, Atlanta, GA 30329, USA; draheja@emory.edu (D.R.); evelyn.p.davila@gmail.com (E.P.D.); ericj51894@gmail.com (E.T.J.); rijalda.deovic@emory.edu (R.D.); m.b.paine@emory.edu (M.P.)

**Keywords:** elderly, recruitment, vaccine clinical trials, willingness to participate

## Abstract

The purpose of this study is to understand among a convenience sample of 400 adults aged 60 years of age or older (1) reasons for being willing or unwilling to participate in a vaccine clinical research study and (2) overall perceptions about vaccine clinical research. A cross-sectional study using a sample of older adults residing in the metro-Atlanta area and surrounding neighborhoods was conducted. The study questionnaire contained 37 questions, including questions about socio-demographics and perceptions about clinical trial processes. Statistical analysis was conducted using logistic regression. The adjusted modeling results indicated that sex, distance to research clinic, and being informed about the research findings played a role in the likelihood of an elderly person participating in a vaccine study. Males were more likely to participate in clinical trials as compared to females (OR: 2.486; CI: 1.042–5.934). Most participants were willing to travel up to 25 miles from the research clinic. Of the respondents, 45% were unlikely to participate if the results of the current trial are not shared. Improving access to clinical trials in terms of distance traveled and ensuring streamlined processes to inform participants about the results of the trial in the future would increase willingness to participate in vaccine clinical trials. The survey could serve as a useful tool for conducting vaccine studies and other clinical trials by understanding the barriers specific to the elderly.

## 1. Introduction

Adults 60 years of age or older are often under-represented in clinical research [1,2,3,4,5,6,7,8]. This under-representation may be because older adults are more likely than young adults to have adverse effects, be lost to follow-up, or discontinue treatment given the presence of multiple chronic conditions, such as arthritis and cardio-metabolic diseases [5,9,10]. However, studies investigating a preventative or therapeutic strategy for the general adult population require the inclusion of older adults to ascertain more accurately the benefits or risks to adults. For example, several vaccine research studies, such as those focusing on pneumococcal disease and *Clostridium difficile* infection, have targeted older adults because of the increased risk of these infections among older adults. Furthermore, the increasingly aging population (Administration of Aging 2015) will require a better understanding of effective therapies for this population and, therefore, greater inclusion of older adults in clinical trials such as vaccine studies. In 2005, a national initiative in the U.S.—the Eliminating Disparities in Clinical Trials (EDICT)—was launched to address the under-representation of older adults in clinical trials [2].

Vaccine trials are imperative for the development of vaccines that can prevent an individual from acquiring a disease or condition and prevent the spread of the condition in the community. In order to successfully include older adults in vaccine clinical research studies, a greater understanding of factors associated with a participant’s willingness to participate is needed. Although literature exists about potential determinants or predictors for the inclusion of older adults into research studies investigating various health topics [10,11,12,13,14,15,16,17,18,19,20,21,22,23,24], most of these have focused on older adults with a specific health condition, such as cancer, or demographic characteristic, such as race. However, participants may be more reluctant to participate in a vaccine clinical trial given the complexity of these trials, perceived risks, and time and effort required from participants.

To our knowledge, no study has focused on factors associated with recruitment of older adults into clinical trials testing vaccines among a general (i.e., no specific demographic characteristic) sample of older adults with relatively good health. The purpose of this study is to understand among a convenience sample of 400 adults aged 60 years of age or older (1) reasons for being willing or unwilling to participate in a vaccine clinical research study and (2) overall perceptions about vaccine clinical research.

## 2. Methods

### 2.1. Study Population

A cross-sectional study was conducted using a convenience sample of 400 older adults at least 60 years of age residing in the metro-Atlanta area and surrounding neighborhoods. To be included in the study, adults had to be 60 years of age or older, able to understand English, and have no apparent or self-disclosed mental or physical impairment preventing them from completing the informed consent and study questionnaire.

### 2.2. Recruitment and Enrollment

Recruitment took place between November 2016–April 2017 in senior housing facilities for low to high income, recreation centers, learning institutes for seniors, and multipurpose senior centers. Two trained study staff visited these facilities on days and times approved by the facilities, which were usually between 9:30 a.m. and 3:00 p.m. on weekdays. Some facilities posted study flyers at their facilities before the study team’s visit. Potential participants approached at the facilities were given the option to complete the informed consent and study questionnaire at home and mail back the documents.

At enrollment, the study questionnaire was given to each participant; if the potential participant was recruited in-person, then the questionnaire was either self-administered or read to them by a trained study staff member. If the potential participant was recruited over the phone, then a trained study staff read the questionnaire to them over the phone or sent the questionnaire to them by mail with a return envelope. Written informed consent was obtained for surveys completed in person and verbal informed consents for surveys completed by phone. A total of 485 participants were approached, of which 85 participants did not give informed consent and refused survey participation.

### 2.3. Staff Training and Data Management

All study staff were trained on the informed consent process, good clinical practices, and human subject protection policies and standards. We obtained approval from the Emory Institutional Review Board before conducting the study. The participants were asked to not include their names or any other personal contact information in the questionnaires. All questionnaires were labeled with an identification number of the participant. All completed surveys and signed informed consents were kept in a locked cabinet at the study office. Data entered in the study database was de-identified. Participants were given a $10 gift card as compensation for completing the study questionnaire.

### 2.4. Study Variables

The study questionnaire contained 37 questions, including questions about socio-demographics; physical health and well-being; and knowledge, beliefs, and concerns about participating in clinical research or about the clinical trial process. The outcome variable was the willingness to participate in a vaccine-related clinical trial (yes versus no). Independent variables considered for this study were those found to be associated with participation in research among older adults in prior research. Most variables were categorical except the variables about beliefs and concerns, which were ordinal, and age, which was continuous. Likert scale was used as an assessment tool to understand participants’ perspectives on vaccine clinical trials. Table 1 includes socio-demographic and health status variables. Socio-demographic variables included age, sex, race, highest grade of school or year of college completed, marital status, employment status, and means of transport to the clinic. Health status variables included health status grading, health limitations, supervised care for chronic conditions, health problems requiring the use of special equipment, use of multiple prescription medications, stress related to buying nutritious meals, and need for emotional support. Table 2 includes variables related to population characteristics and perceptions about clinical trials.

### 2.5. Statistical Analysis

Descriptive of all variables were performed, including mean and standard deviation for age (continuous variable) and count and percentages for all other variables (categorical variables). The association between each of the dependent variable and each of the independent variables was tested using unadjusted binary logistic regression models. Variables found statistically associated with the dependent variables at the 0.05 alpha level were included in the final (adjusted) logistic regression model. Age, sex, and race/ethnicity were included regardless of significance and were included in the model first. All other independent variables were included sequentially, with the demographic variables first. The final (adjusted) logistic regression model included age, sex, race, the highest grade of school or year of college completed, previous participation in a vaccine-related clinical study, being informed in the future about the research, distance to research clinic, health status, and health limitations. Model fit of the adjusted model was assessed using the Hosmer–Lemeshow test. Odds ratio were computed for the variables included in the (adjusted) logistic regression model and a confidence interval of 95% was used. The data analysis for this paper was generated using SAS^®^ software, Version 9.4 of the SAS System. Copyright ©2012–2017 SAS Institute Inc. SAS and all other SAS Institute Inc. product or service names are registered trademarks or trademarks of SAS Institute Inc., Cary, NC, USA. 

## 3. Results

This survey was conducted among 400 participants across a diverse demographic population, including White/Caucasian (60.05%), Black/African American (30.26%), and Hispanic (4.10%) participants. Females formed a majority of participants (69.00%. The mean age of the population was 75.33 ± 8.80. Just over a quarter (25.50%) were currently married, while 32% were widowed. Of the participants, 93.50% had completed high school, while 46.25% had completed college or post-college education. Table 1 lists the socio-demographics and overall health status of participants. Majority of the respondents (64.34%) were willing to participate in vaccine clinical trials; however, 75.00% had never participated in a clinical trial before. Three-quarters (75.00%) were retired from their occupation and most of them used Medicare as health insurance (55.59%). Many respondents (79%) perceived to be in good, very good, or excellent health status. However, 30.33% had limitations due to physical, mental, or emotional problems, and 25.25% had a health problem requiring the use of specialized equipment, such as a cane, a wheelchair, a special bed, or a special telephone. Of the participants, 38.25% were under supervised care from a doctor or healthcare provider for a chronic medical condition, and 74.00% were taking multiple prescription medications. Some 42.00% had been worried or stressed about having enough money to buy nutritious meals at least once in their lifetime. Further details about population characteristics and perceptions about clinical trials among participants are shown in Table 2.

Males were more likely to participate in clinical trials as compared to females (OR: 2.486; 95% CI: 1.042–5.934). Most participants, i.e., 51.75%, were willing to travel up to 25 miles from the research clinic, whereas 24.75% agreed to travel no more than 15 miles, 17.75% would travel no more than 5 miles, and 9.25% would travel no more than 25 miles. Some participants (8.75%) said that they would travel to a research clinic regardless of how far it was. The odds ratio of traveling no more than 5 miles was 2.501 (95% CI: 0.966–6.475), no more than 15 miles was 5.194 (95% CI: 1.913–14.105) and no more than 25 miles was 3.205 (95% CI: 0.774–13.276). In regard to the means of transport, 46.50% drove their vehicle, 20.50% used public transport, 20.25% were dependent on a friend or family member to drive them, 6.75% used hospital transport, 2.00% used taxi or bicycle and the remaining 1.50% traveled by foot. Participants were unlikely to participate if the results of current trial are not shared; odds ratio for participants who agreed/strongly agreed with that statement was 0.253 (95% CI: 0.098–0.653). Whereas 45.25% of participants were unlikely to participate if the results of a current trial were not shared, 26.50% would still participate, and 12.25% were neutral. Table 3 displays odds of willingness to participate in a vaccine study among the sample.

With respect to the source of information regarding clinical trials, 41.50% reported research clinic staff or investigator as the source, 16.75% reported the source as community events or groups, 4.75% through flyers, 3.00% heard about the studies through research clinic website, 3.00% through the radio, and 2.0% through other internet sources. Many (67.50%) were unaware that clinical trials information is publicly accessible at ClinicalTrials.gov. Survey participants with a better health status were more likely to participate in the clinical trial—excellent/very good health OR: 2.451 (95% CI: 0.808–7.431) and good health OR: 2.498 (95% CI: 0.803–7.775).

## 4. Discussion

The elderly differ from younger populations in terms of pharmacodynamics, pharmacokinetics, and study product responses. The International Conference on Harmonisation (ICH) guidelines recommend that a minimum of 100 geriatric patients should be included in Phase III clinical trials for drugs used in diseases not unique to, but present in, elderly populations to demonstrate these clinical differences [25]. Participation of elderly populations in vaccine clinical trials remains a challenge compared to drug clinical trials that offer treatments to ill patients. As a result, very few vaccine clinical trials enroll elderly subjects. Akmatov et al. conducted a survey among participants over the age of 65 for participation in a possible study on influenza vaccinations [26]. Non-participation in the study was mainly associated with the invasive nature of medical study procedures, such as blood-draws, and lengthy amount of time required for participation among other reasons [26].

This study was designed to understand the views and experiences of elderly populations in clinical trial participation, particularly for vaccines. Most respondents were willing to participate in clinical trials and had an overall positive attitude towards participation. The adjusted modeling results indicated that sex, distance to research clinic, and being informed in the future about the research findings played a role in the likelihood of an elderly person participating in a vaccine study. The major barriers to participating in vaccine clinical trials were the distance to the research facility and not being informed about the results of the trial in the future.

Males were most likely to participate in vaccine trials compared to females, which is consistent with the findings of participation in AIDS, colorectal cancer, and lung cancer trials [27,28]. Since males and females differ in pharmacokinetic and pharmacodynamics characteristics, it is important that females are adequately represented in clinical trials. Most frequent reasons for non-participation by women positive for HIV in clinical trials were that they would not be informed about trials, did not want to or were not interested, fear of experimentation, and ineligibility to participate [27].

Less than a quarter of participants were willing to travel 25 miles or would travel irrespective of distance. Since elderly patients have multiple barriers to travel, such as limited mobility due to comorbidities or disabilities, inability to drive, or poor vision, it is expected that longer travel distance for research purposes would be a barrier for participation in clinical trials. Providing free transport to participants would make it easier for them to travel to research sites and perhaps increase willingness to travel longer distances. The mode of transport and availability of public transport could also play a role in deciding the optimum travel distance to the research clinic; less than half of the participants in our study drove their own vehicle. A study conducted by Bruner et al. using Geographic Information System (GIS) maps showed that participants traveled a median of 11.6 miles to participate in clinical trials [29]. It also suggested strategies to improve minority access to cancer clinical trials, including identification of minority rich areas without cooperative group site membership, assessing best practices for recruitment from sites reporting highest minority accrual, and documenting transport issues [29].

Most of the participants were unlikely to participate if the results of the current trial were not shared in the future. Participants are generally curious to know the outcome of trials in which they participate. For ethical reasons, research teams must take the responsibility of informing participants of study results after trial termination. In cases where participants receive experimental vaccines or other drugs/devices, this could also affect their health in future and is mandatory for appropriate unblinding processes. In a study about perspectives of adult patients and parents of pediatric patients undergoing hematopoietic stem cell transplantation, many patients criticized the general lack of feedback [30]. One of the interviewed participants reported not knowing if the trial had ended and would prefer to get a notification about study outcomes [30]. Many participants enroll in trials for altruistic reasons and to contribute to scientific research and prefer to stay informed about the efficacy and results of trials. There are regulatory requirements for submitting results information for certain clinical trials to ClinicalTrials.gov per the Final Rule for Clinical Trials Registration and Results Information Submission (42 CFR Part 11) [31]. As per the International Committee of Medical Journal Editors (ICMJE), one of the conditions to publish clinical trial research results is to register the trial on ClinicalTrials.gov [31].

The research team and investigators play a key role in the recruitment of elderly participants. If the respondent believes that the study result of a vaccine clinical trial or the study will benefit the respondent, they are more likely to participate in the study. This indicates the importance of the research team explaining the benefits and risks to the participants, primarily when the trial provides treatment or disease prevention. Almost an equal number of participants were comfortable in being part of a clinical trial if the vaccine had not been tested in humans before compared to those that were not comfortable with this situation. This re-emphasizes the significance of good consenting practices and explanation of the risk–benefit ratio of vaccines to participants. Not comprehending previous testing procedures is a possible explanation for why it is more difficult to recruit for phase I trials as unexpected side effects are usually not known at this stage. Most participants heard about clinical trials from research clinic staff or investigators, while the least number of participants reported the source of information as clinic websites or other internet sources, probably due to lack of technological knowledge and lesser use of internet resources among the elderly. Directly reaching out to this population via phone or in person at clinics or healthcare events seems to be a more successful method of recruitment as compared to internet advertisements.

The study had a few limitations. Firstly, it was conducted in metro-Atlanta area and surrounding neighborhoods, which limits the generalizability of our findings. Another limitation was the small sample size, which could potentially increase variability and bias. The survey questionnaire did not specify the specific type of vaccine or disease or phase of the trial, the response to this questionnaire and willingness to participate could be different if a specific scenario was described to participants. Lastly, this was a highly structured questionnaire, and there may be other factors that contribute to a person’s willingness to participate that were not captured in the survey. Additional research in this area may be needed to further understand the barriers of elderly participation in vaccine clinical trials.

## 5. Conclusions

The survey suggests that improving access to clinical trials—in terms of distance traveled and ensuring streamlined processes to inform participants about the results of the trial in the future—would increase their willingness to participate in vaccine clinical trials. The survey could serve as a useful tool for implementing studies that recruit elderly population, and data can be subsequently used to improve recruitment and participant experience in vaccine clinical trials specifically, and drug/device clinical trials in general.

## Figures and Tables

**Table 1:** Socio-demographics and overall health status among 400 participants.

Variable	Mean	Standard Deviation
**Age**	75.33	8.80
	**Number of Respondents (*N*)**	**Respondents (%)**
**Sex**		
Male	108	27.00
Female	276	69.00
**Race**		
Non-Hispanic White/Caucasian	242	60.05
Black/African American	118	30.26
Hispanic	16	4.10
Other	10	2.56
**Highest grade of school or year of college completed**		
Grades 1–11	26	6.50
High school	75	18.75
Some college	103	25.75
College graduate	80	20.00
Post-college	105	26.25
**Marital status**		
Married	102	25.50
Partner	5	1.25
Separated	17	4.25
Divorced	97	24.25
Widowed	128	32.00
Never married	36	9.00
**Health Status**		
Excellent	50	12.50
Very Good	119	29.75
Good	147	36.75
Fair	65	16.25
Poor	7	1.75
**Have limitations due to physical, mental, or emotional problems**		
Yes	118	30.33
No	248	63.75
Don’t know	19	4.88
Refuse	4	1.03
**Have a health problem requiring the use of special equipment, such as a cane, a wheelchair, a special bed, or a special telephone**		
Yes	101	25.25
No	288	72.00
**Under supervised care from a doctor or healthcare provider for a chronic medical condition**		
Yes	153	38.25
No	220	55.00
**Taking multiple (more than one) prescription medications**		
Yes	296	74.00
No	81	20.25
**In the past 12 months, have been worried or stressed about having enough money to buy nutritious meals.**		
Always	17	4.25
Usually	12	3.00
Sometimes	68	17.00
Rarely	58	14.50
Never	232	58.00
**Frequency in getting needed social and emotional support.**		
Always	148	37.00
Usually	141	35.25
Sometimes	52	13.00
Rarely	23	5.75
Never	19	4.75
Type of health insurance *		
Private health	101	17.12
Medicare	328	55.59
Medicaid	67	11.36
Military Health	21	3.56
Other	70	11.86
No or health care coverage	3	0.51
**Employment status**		
Working now	42	10.50
Unemployed and looking for work	14	3.50
Disabled	24	6.00
Retired	300	75.00
Homemaker	3	0.75
Other	10	2.50
**Means of transportation to medical appointments**		
By public transportation	82	20.50
A friend or relative drives me	81	20.25
By hospital transport	27	6.75
I drive myself	186	46.50
On foot	6	1.50
By taxi	5	1.25
By bicycle	3	0.75

Note: Numbers do not add up to 100% because “don’t know” and “refuse” responses were removed from the table but included during analysis. * Some participants had multiple types of health insurance, hence the total exceeds sample size.

**Table 2:** Population characteristics and perceptions about clinical trials among 400 participants.

Variable	Number of Respondents (*N*)	Respondents (%)
**Willingness to participate**		
Yes	249	62.25
No	126	31.50
**Previous Participation in a vaccine related clinical study**		
Yes	79	19.75
No	300	75.00
**Participant’s spouse, family, or close friends need(s) to feel comfortable with subject participation**		
Strongly agree	59	14.75
Agree	104	26.00
Neutral	59	14.75
Disagree	88	22.00
Strongly disagree	39	9.75
**Physician/healthcare provider needs to be comfortable with subject participation**		
Strongly agree	82	20.50
Agree	139	34.75
Neutral	58	14.50
Disagree	48	12.00
Strongly disagree	21	5.25
**The study result of a vaccine clinical trial or study will benefit the participant**		
Strongly agree	65	16.25
Agree	159	39.75
Neutral	103	25.75
Disagree	25	6.25
Strongly disagree	4	1.00
**The study result of a vaccine clinical trial or study will benefit others or will contribute to scientific research**		
Strongly Agree	142	35.50
Agree	189	47.25
Neutral	25	6.25
Disagree	4	1.00
Strongly disagree	0	0.00
**It is acceptable being placed ‘at random’ in either study group.**		
Strongly Agree	99	24.75
Agree	192	48.00
Neutral	42	10.50
Disagree	16	4.00
Strongly disagree	6	1.50
**Interest in study participation despite potential side effects**		
Strongly Agree	24	6.00
Agree	75	18.75
Neutral	85	21.25
Disagree	74	18.50
Strongly disagree	30	7.50
**Comfortable participating in a vaccine clinical trial that has never been tested in human beings.**		
Strongly Agree	26	6.50
Agree	92	23.00
Neutral	74	18.50
Disagree	81	20.25
Strongly disagree	31	7.75
**Unlikely to participate in future trials, if results of current trial are not shared**		
Strongly Agree	46	11.50
Agree	135	33.75
Neutral	49	12.25
Disagree	78	19.50
Strongly disagree	28	7.00
**Reasonable safety precautions likely to be taken in a clinical trial**		
Strongly Agree	109	27.25
Agree	200	50.00
Neutral	27	6.75
Disagree	10	2.50
Strongly disagree	4	1.00
**All possible measures to protect privacy are likely to be taken a clinical trial**		
Strongly Agree	149	37.25
Agree	180	45.00
Neutral	24	6.00
Disagree	4	1.00
Strongly disagree	1	0.25
**Most of the current treatments in medicine are based on evidence from clinical trials**		
Strongly Agree	91	22.75
Agree	185	46.25
Neutral	38	9.50
Disagree	12	3.00
Strongly disagree	2	0.50
**The typical monetary compensation for participating in a clinical trial is enough for time and travel**		
Strongly Agree	58	14.50
Agree	208	52.00
Neutral	46	11.50
Disagree	24	6.00
Strongly disagree	9	2.25
**The typical amount of time needed for participation in a clinical trial is feasible and acceptable**		
Strongly Agree	49	12.25
Agree	222	55.50
Neutral	42	10.50
Disagree	26	6.50
Strongly disagree	6	1.50
**Source of information regarding clinical trials**		
Research Clinic Staff or Investigator	166	41.50
Research Clinic website	12	3.00
Radio	12	3.00
Flyer	19	4.75
Internet other than Hope Clinic Website or Facebook	8	2.00
Community events or group	67	16.75
Other	76	19.00
**Distance (in miles) willing to drive to a research clinic for a vaccine clinical trial**		
No more than 5 miles	71	17.75
No more than 15 miles	99	24.75
No more than 25 miles	37	9.25
It does not matter how many miles, I would still go the research clinic	35	8.75
Not willing to drive any miles	87	21.75
**Aware that clinical trials information can be accessed at ClinicalTrials.gov**		
Yes	72	18.00
No	270	67.50
**Military service**		
Yes	56	14.00
No	339	84.75
**Occupation in the public health, healthcare, or scientific research field**		
Yes	91	22.75
No	257	64.25
**Means of transportation to medical appointments**		
By public transportation	82	20.50
A friend or relative drives me	81	20.25
By hospital transport	27	6.75
I drive myself	186	46.50
On foot	6	1.50
By taxi	5	1.25
By bicycle	3	0.75

Note: Numbers do not add up to 100% because “don’t know” and “refuse” responses were removed from the table but included during analysis.

**Table 3:** Odds of willingness to participate in a vaccine study among adults 60 years of age or older, Metro-Atlanta, GA, 2016.

Variable	Odds Ratio (95% CI)	
	Unadjusted Model	Adjusted Model
**Age**		
60–65	1.604 (0.728–3.533)	0.855 (0.174–4.203)
66–75	1.171 (0.727–1.885)	0.723 (0.312–1.677)
>76	Reference	Reference
**Sex**		
Male	3.131 (1.777–5.517)	2.486 (1.042–5.934)
Female	Reference	Reference
**Race**		
Non-Hispanic White/Caucasian	Reference	Reference
Black/African American	1.054 (0.647–1.717)	0.492 (0.072–3.339)
Hispanic	0.866 (0.304–2.470)	0.662 (0.086–5.117)
Other	0.780 (0.214–2.844)	0.328 (0.026–4.064)
**Highest grade of school or year of college completed**		
Grades 1–11	Reference	Reference
High school	0.708 (0.271–1.852)	1.486 (0.189–11.674)
Some college	0.875 (0.343–2.229)	1.631 (0.234–11.395)
College graduate	0.981 (0.375–2.569)	1.635 (0.227–11.747)
Post-college	0.931 (0.366–2.366)	0.919 (0.130–6.480)
**Previous participation in a vaccine related clinical study**		
Yes	6.765 (3.003–15.240)	9.567 (1.944–47.094)
No	Reference	Reference
**Unlikely to participate in future trials, if results of current trial are not shared**		
Strongly Agree or agree	0.282 (0.154–0.515)	0.253 (0.098–0.653)
Neutral	0.822 (0.336–2.013)	0.622 (0.155–2.493)
Strongly disagree or disagree	Reference	Reference
**Distance (in miles) willing to drive to a research clinic for a vaccine clinical trial**		
No more than 5 miles	1.777 (0.928–3.404)	2.501 (0.966–6.475)
No more than 15 miles	4.706 (2.412–9.180)	5.194 (1.913–14.105)
No more than 25 miles	5.683 (2.147–15.043)	3.205 (0.774–13.276)
It does not matter how many miles, I would still go the research clinic	--	--
Not willing to drive any miles	Reference	Reference
**Health Status**		
Excellent or very good	3.008 (1.675–5.403)	2.451 (0.808–7.431)
Good	3.355 (1.823–6.175)	2.498 (0.803–7.775)
Fair or poor	Reference	Reference
**Have limitations due to physical, mental, or emotional problems**		
Yes	0.530 (0.332–0.846)	0.615 (0.264–1.432)
No	Reference	Reference

Note: Hosmer–Lemeshow test was applied to assess the goodness of fit of the model, and *p* > 0.05 indicated a good fit (χ^2^ = 5.92; df = 8; *p* = 0.66).
